# Synthesis and evaluation of antimicrobial, antitubercular and anticancer activities of 2-(1-benzoyl-1*H*-benzo[*d*]imidazol-2-ylthio)-*N*-substituted acetamides

**DOI:** 10.1186/s13065-018-0432-3

**Published:** 2018-05-26

**Authors:** Snehlata Yadav, Siong Meng Lim, Kalavathy Ramasamy, Mani Vasudevan, Syed Adnan Ali Shah, Abhishek Mathur, Balasubramanian Narasimhan

**Affiliations:** 1 Faculty of Pharmaceutical Sciences, Maharshi Dayanand University, Rohtak, 124001 India; 2 Faculty of Pharmacy, Universiti Teknologi MARA (UiTM), 42300 Bandar Puncak Alam, Selangor Darul Ehsan Malaysia; 3 Collaborative Drug Discovery Research (CDDR) Group, Pharmaceutical Life Sciences, Community of Research, Universiti Teknologi MARA (UiTM), 40450 Shah Alam, Selangor Darul Ehsan Malaysia; 4 Department of Pharmacology and Toxicology, College of Pharmacy, Qassim University, Buraidah, 51452 Saudi Arabia; 5 Atta-ur-Rahman Institute for Natural Products Discovery (AuRIns), Universiti Teknologi MARA, Puncak Alam Campus, 42300 Bandar Puncak Alam, Selangor Darul Ehsan Malaysia; 6 Rapture Biotech, Noida, India

**Keywords:** MCF7, HCT116, Isocitrate lyase, Pantothenate synthetase, Resistance, Cytotoxic, In vitro

## Abstract

**Background:**

The study describes the synthesis, characterization, in vitro antimicrobial and anticancer evaluation of a series of 2-(1-benzoyl-1*H*-benzo[*d*]imidazol-2-ylthio)-*N*-substituted acetamide derivatives. The synthesized derivatives were also assessed for in vitro antitubercular activity against *Mycobacterium tuberculosis* H37Rv. The compounds found active in in vitro study were assessed for their in vivo antitubercular activity in mice models and for their inhibitory action on vital mycobacterial enzymes viz, isocitrate lyase, pantothenate synthetase and chorismate mutase.

**Results:**

Compounds **8**, **9** and **11** emerged out as excellent antimicrobial agents in antimicrobial assays when compared to standard antibacterial and antifungal drugs. The results of anticancer activity displayed that majority of the derivatives were less cytotoxic than standard drugs (tamoxifen and 5-fluorouracil) towards MCF7 and HCT116 cell lines. However, compound **2** (IC_50_ = 0.0047 µM/ml) and compound **10** (IC_50_ = 0.0058 µM/ml) showed highest cytotoxicity against MCF7 and HCT116 cell lines, respectively. The results of in vivo antitubercular activity revealed that a dose of 1.34 mg/kg was found to be safe for the synthesized compounds. The toxic dose of the compounds was 5.67 mg/kg while lethal dose varied from 1.81 to 3.17 mg/kg body weight of the mice. Compound **18** inhibited all the three mycobacterial enzymes to the highest level in comparison to the other synthesized derivatives but showed lesser inhibition as compared to streptomycin sulphate.

**Conclusions:**

A further research on most active synthesized compounds as lead molecules may result in discovery of novel anticancer and antitubercular agents.

## Background

In the twentieth century, greatest advances have been made to tackle microbial infections in human beings. However, the problem of developing resistance to the existing antimicrobial agents has become a nuisance for the medical professionals as the microbes have become capable of evading from the lethal action of most these agents [1]. Tuberculosis (TB) is a contagious disease caused by omnipresent mycobacteria i.e., *Mycobacterium tuberculosis* [2]. According to 2015 survey of WHO, the world had an estimated 10.4 million new TB cases. TB is one of the biggest killers striking people in their most productive years and accounts for 23% of the global TB burden in India alone [3]. The synergy of this disease with HIV infection and; emergence of multidrug resistance and extensively drug resistance tuberculosis (MDRTB and XDRTB) to the first-line drugs are the threatening global challenges [4]. The researchers have left no stone unturned to discover lead molecules against the disease even then no new chemical entity has appeared for use in clinical treatment of this disease over the last four decades [5].

Cancer, the most debilitating disease, has advanced to such a level that it has become one of the universal cause of human suffering and death all over the world [6, 7]. The huge arsenal of synthetic, semi-synthetic, and naturally-occurring agents for treating neoplastic diseases suffers from two major limitations; the first one being the lack of selectivity of conventional chemotherapeutic agents to cancer tissues, causing unwanted side effects [8]. The second is the acquisition of multiple-drug resistance by cancer cells to the available agents that impedes treatment of various kinds of cancer [9]. Therefore, developing novel molecules to circumvent multidrug resistances and exhibiting selective toxicity to cancer cells rather than to normal cells is need of the hour.

Heterocycles are of considerable interest to the researchers in the field of medicinal chemistry [10]. Benzimidazole is present in several natural and synthetic medicinal compounds and hence is most comprehensively studied bioactive heterocycle [11]. The broad-spectrum biological profile of benzimidazole derivatives includes, hormone antagonist [12], anti-HIV [13, 14], anthelmintic [15], antiprotozoal [16], antihypertensive [17], antioxidant, anti-inflammatory [18], analgesic [19], anxiolytic [20], anticoagulant [21], antifungal [22], antihistaminic [23], antiulcer [24], anti-obesity, antidiabetic [25], antimicrobial [26], antimycobacterial [27] and anticancer [28, 29] activities. In the light of above facts and in continuation of efforts in developing novel molecules for the treatment of tuberculosis and cancer [30, 31], in the present study we herein report the synthesis, antimicrobial, anticancer and antitubercular activities of benzimidazole derivatives i.e., 2-(1-benzoyl-1*H*-benzo[*d*]imidazol-2-ylthio)-*N*-substituted acetamides.

## Results and discussion

### Chemistry

**Scheme 1:**
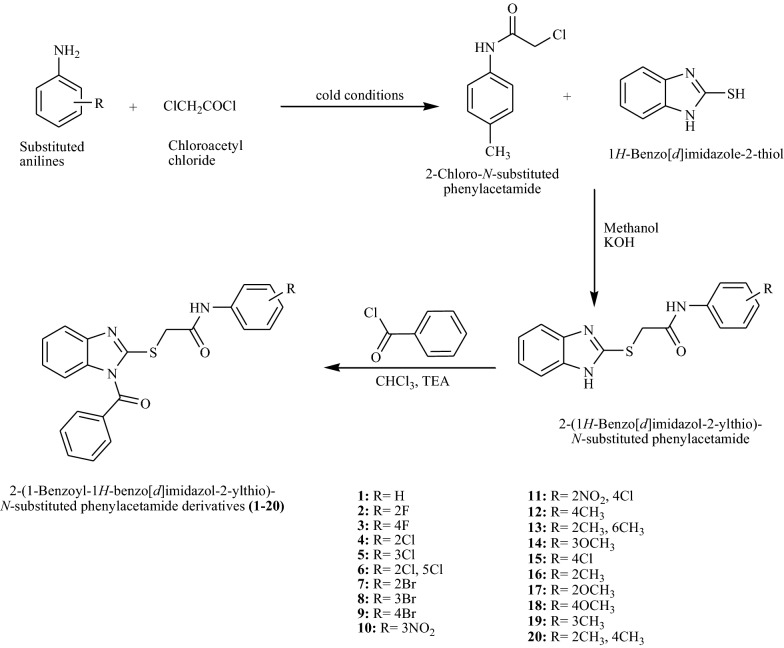
Synthesis of 2-(1-benzoyl-1*H*-benzo[*d*]imidazole-2-ylthio)-2-ylthio)-*N*-substituted acetamide derivatives (**1**–**20**)

2-(1-Benzoyl-1*H*-benzo[*d*]imidazole-2-ylthio)-2-ylthio)-*N*-substituted acetamide derivatives (**1**–**20**) were synthesized according to Scheme 1 and characterized by physicochemical and spectral means. The structures of obtained compounds (**1**–**20**) were confirmed by IR, ^1^HNMR, ^13^CNMR and mass spectroscopic data which was consistent with the proposed molecular structures. The appearance of C=O stretch in the range of 1670–1630 cm^−1^ and N–H stretch 3350–3100 cm^−1^ of secondary amide indicated the formation of secondary amide in the synthesized compounds. The presence of methyl in compound **13**, **16**, **19** and **20** was demonstrated by the presence of CH stretch at 3103 cm^−1^. The multiplet corresponding to 7.14–7.78 δ ppm confirmed the presence of protons of benzimidazole and aryl nucleus. A singlet at around δ 3.8 ppm corresponded to the protons of the methylene in the synthesized compounds.

#### In vitro antimicrobial activity

The results of in vitro antimicrobial activity of the synthesized compounds are presented in Table 1. The synthesized compounds were found to be highly efficient antimicrobial agents in comparison to the standard drug cefadroxil and fluconazole. Amongst the synthesized derivatives, compounds **7**, **8**, **9** and **11** were found to be highly potent antibacterial agents against Gram positive as well as Gram negative bacterial species with MIC of 0.027 µM/ml for each. Compound **7** (MIC = 0.027 µM/ml) showed activity against *Aspergillus niger* also. Compounds **8**, **9** and **11** were highly active towards *Candida albicans* and *A. niger* than the standard antifungal drug fluconazole. The results of minimum bactericidal concentration/minimum fungicidal concentration (Table 2) conveyed that none of synthesized derivatives was either bactericidal or fungicidal in action (In general, a compound is said to be bactericidal/fungicidal if its MBC/MFC is less than three times of its MIC) [32].

**Table 1:** Antimicrobial (MIC = µM/ml) and anticancer (IC_50_ = µM/ml) screening results of 2-(1-benzoyl-1*H*-benzo[*d*]imidazole-2-ylthio)-2-ylthio)-*N*-substituted acetamides

Comp. no.	Microbial strains							Cancer cell lines	
	*S. aureus*	*B. cereus*	*B. subtilis*	*S. typhi*	*E. coli*	*C. albicans*	*A. niger*	MCF7	HCT116
1	0.032	0.032	0.032	0.032	0.032	0.032	0.032	0.0774	> 0.2581
2	0.031	0.031	0.031	0.031	0.031	0.015	0.031	0.0047	0.0839
3	0.031	0.031	0.031	0.031	0.031	0.015	0.031	0.0247	0.1973
4	0.030	0.030	0.030	0.030	0.030	0.015	0.030	0.0356	0.0594
5	0.030	0.030	0.030	0.030	0.030	0.015	0.030	0.0831	0.1307
6	0.027	0.027	0.027	0.027	0.027	0.014	0.027	0.0898	0.0833
7	0.027	0.027	0.027	0.027	0.027	0.027	0.027	0.0558	0.0965
8	0.027	0.027	0.027	0.027	0.027	0.013	0.027	0.0686	0.1072
9	0.027	0.027	0.027	0.027	0.027	0.013	0.027	> 0.2144	0.0643
10	0.029	0.029	0.029	0.029	0.029	0.015	0.029	0.0786	0.0058
11	0.027	0.027	0.027	0.027	0.027	0.013	0.027	0.1606	0.0236
12	0.031	0.031	0.031	0.031	0.031	0.016	0.031	0.1245	0.0398
13	0.030	0.030	0.030	0.030	0.030	0.015	0.030	0.1348	0.0963
14	0.030	0.030	0.030	0.030	0.030	0.030	0.030	0.0958	0.0958
15	0.030	0.030	0.030	0.059	0.030	0.030	0.030	0.0950	0.0594
16	0.031	0.031	0.031	0.031	0.031	0.016	0.031	0.1245	0.0872
17	0.030	0.030	0.030	0.030	0.030	0.015	0.030	–	–
18	0.030	0.030	0.030	0.030	0.030	0.015	0.030	–	–
19	0.031	0.031	0.031	0.031	0.031	0.016	0.031	–	–
20	0.030	0.030	0.030	0.030	0.030	0.015	0.030	–	–
Cefadroxil	0.37	0.37	0.37	0.37	0.37	–	–	–	–
Fluconazole	–		–		–	0.47	0.47	–	–
5-FU	–	–	–	–	–	–	–	–	0.0125
Tamoxifen	–	–	–	–	–	–	–	0.0043	–

**Table 2:** MBC/MFC (µg/ml) of 2-(1-benzoyl-1*H*-benzo[*d*]imidazole-2-ylthio)-2-ylthio)-*N*-substituted acetamides

Comp. no.	MBC (µg/ml)					MFC (µg/ml)	
	*S. aureus*	*B. cereus*	*B. subtilis*	*S. typhi*	*E. coli*	*C. albicans*	*A. niger*
1	50	> 50	> 50	50	50	50	> 50
2	> 50	> 50	50	50	50	50	> 50
3	> 50	> 50	> 50	> 50	50	25	> 50
4	50	> 50	> 50	50	50	25	> 50
5	50	> 50	50	50	50	50	> 50
6	> 50	> 50	> 50	25	> 50	50	> 50
7	50	> 50	> 50	> 50	50	50	> 50
8	50	> 50	> 50	> 50	> 50	50	> 50
9	> 50	> 50	> 50	50	> 50	50	> 50
10	50	> 50	50	50	> 50	> 50	> 50
11	50	> 50	50	50	50	> 50	> 50
12	> 50	> 50	> 50	50	50	25	> 50
13	50	> 50	> 50	> 50	50	25	> 50
14	> 50	> 50	> 50	> 50	50	50	> 50
15	50	> 50	> 50	> 50	50	> 50	> 50
16	50	> 50	50	50	> 50	25	> 50
17	50	> 50	> 50	> 50	> 50	50	> 50
18	> 50	> 50	> 50	> 50	50	50	> 50
19	50	> 50	50	> 50	> 50	50	> 50
20	50	> 50	50	50	> 50	25	> 50

#### In vitro antitubercular activity

The synthesized benzimidazole derivatives were evaluated for their in vitro antitubercular activity against *Mycobacterium tuberculosis* H37Rv (NCFT/TB/537). The zone of inhibition as well as MIC values of the test compounds was determined. Minimum lethal concentration (MLC) of the compounds was also determined. The results of in vitro antitubercular activity are presented in Table 3.

**Table 3:** Antimycobacterial activity, MIC and MLC of synthesized derivatives against *M. tuberculosis* H37Rv

Compound no.	Diameter of zone of inhibition (mm) against H37Rv (NCFT/TB/537)	MIC (µg/ml)	MLC (µg/ml)
1	> 20	12.5	25
2	> 20	12.5	25
3	> 20	12.5	25
4	> 20	12.5	25
5	08	17.8	28.12
6	> 20	12.5	25
7	10	15	28
8	> 20	12.5	25
9	08	17.8	28.12
10	> 20	12.5	25
11	10	15	28
12	> 20	12.5	25
13	> 20	12.5	25
14	NA	NA	NA
15	> 20	12.5	25
16	10	15	28
17	> 20	12.5	25
18	> 20	12.5	25
19	NA	NA	NA
20	10	15	28
Streptomycin	> 20	12.5	25

*NA* no activity

#### In vivo antitubercular activity

The LD_50_ and ED_50_ were determined for the active compounds in mice models infected with *Mycobacterium* H37Rv (Table 4). It was found that the toxic dose of the compounds which proved fatal and highly toxic to mice was 5.67 mg/kg while LD_50_ varied from 1.81 to 3.17 mg/kg body weight of the mice. LD_50_ is the dose that killed 50% of the mice population within the group. Thus, ED_50_ of 1.34 mg/kg was considered safe for each of the compounds. It was observed that this dose was effective and safe for mice in different groups before infecting the mice models with specific TB bacteria as no mortality of any single animal was recorded.

**Table 4:** Lethal dose (in mg/kg) and percent inhibition of enzymes in Mycobacterium H37Rv groups after treatment with effective dose of 1.34 mg/kg of potent compounds and 25 µg/kg of positive control

Potent compounds	LD_50_ dose (mg/kg)	Percent inhibition of enzyme		
		*M.* ICL activity (IU/L)	*M.* PS activity (IU/L)	*M.* CM activity (IU/L)
1	1.83	62.21	56.34	48.32
2	1.81	58.78	50.13	48.56
3	1.87	52.56	47.56	42.24
4	1.96	48.45	38.45	31.78
6	1.86	56.78	50.65	48.23
8	1.88	59.45	42.21	37.45
10	1.83	61.23	54.32	42.23
12	1.85	43.45	36.45	28.76
13	2.14	56.45	54.12	40.23
15	2.67	61.56	56.34	40.34
17	3.17	43.45	36.45	28.76
18	3.15	64.56	60.12	58.23
Negative control	–	No reduction	No reduction	No reduction
Streptomycin sulphate	–	75.12	77.06	79.56

### Mycobacterial enzyme assays

The results of mycobacterial enzyme assays were expressed in terms of percent inhibition of mycobacterial enzymes i.e., isocitrate lyase, pantothenate synthetase and chorismate mutase, by the *M. tuberculosis* H37Rv. The tested compounds inhibited the enzyme activity to a lesser extent that of streptomycin sulphate used as positive control (Table 4). However, compound **18** emerged as the best inhibitor of mycobacterial isocitrate lyase, pantothenate synthetase and chorismate mutase activity showing percentage inhibition of 64.56, 60.12 and 58.23% respectively which was comparable to percent inhibition of 75.12, 77.06 and 79.56% respectively of these enzymes by streptomycin sulphate.

#### In vitro anticancer activity

Almost all the synthesized compounds showed less cytotoxicity towards MCF7 and HCT116 cell lines in comparison to tamoxifen and 5-fluorouracil used as drugs for comparison against MCF7 and HCt116 cell lines, respectively (Table 1). However, compound **2** (IC_50_ = 0.0047 µM/ml) showed almost equal cytotoxicity to tamoxifen (IC_50_ = 0.0043 µM/ml) against MCF7 cell line. On the other hand, compound **10** (IC_50_ = 0.0058 µM/ml) was twice more cytotoxic against HCT116 cell line as compared to 5-fluorouracil (IC_50_ = 0.0125 µM/ml).

### Structure activity relationship

1. Electron withdrawing group fluoro at *ortho* and *para* positions (compounds **2** and **3**) while nitro group at *meta* position (compound **10**) improved anticancer activity. The presence of other electron withdrawing groups like Cl, Br at *ortho*, *meta* or *para* positions diminished the anticancer activity.
2. It is also important to note that fluoro group at position-2 and nitro group at position-3 are essential requirements for anticancer activity.
3. Electron donating groups methoxy and methyl at *para* position (compound **18** and **12**, respectively) have more activating influence on antitubercular activity as compared to *ortho*- and *meta*-positions of these groups and followed the order *p* > *o* > *m.*
4. In general, substitution of electron withdrawing groups like Cl, Br, NO_2_ etc. on the benzene ring has an activating influence on antimicrobial activity while substitution of electron releasing groups like OCH_3_, CH_3_ etc. decreases the antimicrobial activity.

## Conclusion

A series of 2-(1-benzoyl-1*H*-benzo[*d*]imidazole-2-ylthio)-2-ylthio)-*N*-substituted acetamides was synthesized and assessed for its in vitro antimicrobial and anticancer activity against human breast cancer (MCF7) and colorectal (HCT116) cell line. The compounds were also assessed for their in vitro and in vivo antitubercular activity in *M. tuberculosis* H37Rv. The in vivo antitubercular evaluation in mice models infected with *M. tuberculosis* revealed 5.67 mg/kg to be the toxic dose of the compounds that proved fatal and highly toxic to mice while LD_50_ varied from 1.81 to 3.17 mg/kg body weight of the mice. A dose 1.34 mg/kg was found to be safe for each of the compounds. The compounds found to be active in in vivo evaluation were further assessed for their capacity to inhibit the mycobacterial enzymes viz., isocitrate lyase, pantothenate synthetase and chorismate mutase. The tested compounds inhibited these enzymes to a lesser extent than streptomycin sulphate used as positive control. However, compound **18** inhibited the mycobacterial isocitrate lyase, pantothenate synthetase and chorismate mutase activity to 64.56, 60.12 and 58.23% respectively as compared to inhibition of 75.12, 77.06 and 79.56%, respectively by streptomycin sulphate. Compounds **8**, **9** and **11** emerged out as excellent antimicrobial agents in antimicrobial assays when compared to standard antibacterial and antifungal drugs. The results of anticancer activity displayed that majority of the derivatives were less cytotoxic towards MCF7 and HCT116 cell lines when compared with standard drugs tamoxifen and 5-fluorouracil respectively. However, compound **2** (IC_50_ = 0.0047 µM/ml) and compound **10** (IC_50_ = 0.0058 µM/ml) showed highest inhibition against MCF7 and HCT116 cell lines respectively.

## Experimental

### Materials and method

The reagents and chemical used for research work were of analytical grade obtained from commercial sources and used as such without further purification. Melting points were determined by open glass capillary method and are uncorrected. Media and Microbial type cell cultures (MTCC) for antimicrobial activity were obtained on order from Hi-media Laboratories and IMTECH, Chandigarh, respectively. Infrared (IR) spectra was recorded on Bruker 12,060,280, Software: OPUS 7.2.139.1294 spectrophotometer by KBr pellet method and expressed in cm^−1^. The proton nuclear magnetic resonance (^1^H NMR) and carbon nuclear magnetic resonance (^13^CNMR) spectra were traced in deuterated DMSO on Bruker Avance III 600 NMR spectrometer at a frequency of 600 and 150 MHz respectively downfield to tetramethylsilane standard and recorded as chemical shifts in δ ppm (parts per million). The progress of reaction was confirmed by TLC performed on silica gel-G plates and the spots were visualized in iodine chamber. The LCMS data were recorded on Waters Q-TOF micromass (ESI–MS), at Panjab University, India. Elemental analysis for synthesized derivatives was performed on CHNN/CHNS/O analyzer (Flash EA1112N series, Thermo finnigan, Italy).

## Synthesis

### General procedure for synthesis of 2-chloro-*N*-substituted acetamide

An appropriate aniline (0.025 mol) and chloro acetyl chloride (0.037 mol) were separately dissolved in 10 ml of glacial acetic acid and poured into a round bottom flask. The mixture was heated on a water bath with an air condenser till the evolution of hydrochloride gas ceases. The mixture was then cooled to an ambient temperature and about 35 ml of 0.4 M sodium acetate solution was added to it. Thick precipitate so formed was filtered and washed with cold water.

### General procedure for synthesis of 2-(1*H*-benzo[*d*]imidazol-2-ylthio)-*N*-substituted acetamide

Equimolar (0.01 mol) quantities of 2-mercaptobenzimidazole and potassium hydroxide were dissolved in 100 ml of methanol by stirring and simultaneously heating to 50–60 °C. 2-Chloro-*N*-substituted-acetamide (0.01 mol) was added in small lots to the stirred mixture maintaining the temperature of the mixture at 50–60 °C. The reaction mixture was then stirred at room temperature for 12 h and then was poured into ice cold water and stirred for 30 min maintaining the temperature at 5–10 °C. The precipitate formed was filtered, washed with cold water, dried and recrystallized with ethanol.

### General procedure for synthesis of 2-(1-benzoyl-1*H*-benzo[*d*]imidazol-2-ylthio)-*N*-substituted acetamide derivatives (1–20)

To a round bottom flask containing 2-(1*H*-benzo[*d*]imidazol-2-ylthio)-*N*-substituted acetamide (0.01 mol) in about 40 ml of chloroform, 1.4 ml of benzoyl chloride (0.012 mol) and 1.66 ml of triethylamine (0.012 mol) were added. The reaction mixture was refluxed for an appropriate time. The formation of product was confirmed by TLC. The solvent was distilled off and the residue obtained was washed with water, dried and recrystallized with hexane.

### Spectral data of 2-(1-benzoyl-1*H*-benzo[*d*]imidazol-2-ylthio)-*N*-substituted acetamide derivatives (1–20)

#### *2*-*(1*-*Benzoyl*-*1H*-*benzo[d]imidazol*-*2*-*ylthio)*-*N*-*acetamide***(1)**

Light brown crystals, yield 76%, mp 97–100 °C, R_f_ 0.60 (*n*-hexane:ethylacetate 6:4); IR (ν_max_, cm^−1^): 3466 N–H str. for 2° amide, 3069 N–H str. of imidazole, 1702 C=O str for 2° amide, 754 C–S str. of thiol. ^1^H NMR: δ_H_ 3.80 (s, 2H of methylene), 7.06–7.97 (m, 14H, aromatic), 10.87 (s, NH of 2° amide). ^13^C NMR: δ_c_ 36.70 CH_2_ aliphatic, (124.95, 126.07, 127.54, 128.48, 128.50, 128.74, 129.20, 130.70, 131.34, 131.79, 132.80) C of benzene, (113.13, 119.20, 123.70, 138.56, 150.17) C of benzimidazole, 142.5 CH aliphatic, 164.79 C of ketone, 167.25 C of amide. ESI–MS (m/z) [M+1]^+^ 388.36; Anal. Calcd. for C_22_H_17_N_3_O_2_S: C, 68.20; H, 4.42; N, 10.85; O, 8.26; S, 8.28 Found: C, 68.22; H, 4.39; N, 10.82; O, 8.25; S, 8.23.

#### *2*-*(1*-*Benzoyl*-*1H*-*benzo[d]imidazol*-*2*-*ylthio)*-*N*-*(2*-*fluorophenyl)acetamide***(2)**

Light brown, yield 69%, mp 115–118 °C, R_f_ 0.69 (*n*-hexane:ethylacetate 6:4); IR (ν_max_, cm^−1^): 3445 N–H str. for 2° amide, 3046 N–H str. for imidazole, 1695 C=O str. for 2° amide, 1024 C–F str. of monofluorinated compound, 739 C–S str. of thiol. ^1^H NMR: δ_H_ 4.36 (s, 2H of methylene), 7.09–7.97 (m, 13H, aromatic), 10.38 (s, NH of 2° amide. ^13^C NMR: δ_c_ 36.86 CH_2_ aliphatic, (118.17, 122.89, 124.31, 125.26, 125.97, 126.04, 128.37, 129.30, 130.73, 132.62, 133.84, 166.49) C of benzene, (115.48, 123.53, 130.73, 143.00, 152.52) C of benzimidazole, 142.5 CH aliphatic, 167.24 C of ketone, 169.28 C of amide. ESI–MS (m/z) [M+1]^+^ 406.23; Anal. Calcd. for C_22_H_16_FN_3_O_2_S: C, 65.17; H, 3.98; F, 4.69; N, 10.36; O, 7.89; S, 7.91 Found: C, 65.19; H, 3.92; F, 4.66; N, 10.39; O, 7.83; S, 7.87.

#### *2*-*(1*-*Benzoyl*-*1H*-*benzo[d]imidazol*-*2*-*ylthio)*-*N*-*(4*-*fluorophenyl)acetamide***(3)**

Cream colored crystals, yield 75%, mp 182–185 °C, R_f_ 0.80 (*n*-hexane:ethylacetate 6:4); IR (ν_max_, cm^−1^): 3439 N–H str. for 2° amide, 3056 N–H str. for imidazole, 1652 C=O str for 2° amide, 1156 C–F str. of monofluorinated compound, 744 C–S str. of thiol. ^1^H NMR: δ_H_ 4.65 (s, 2H of methylene), 7.14–7.72 (m, 13H, aromatic), 10.98 (s, NH of 2° amide). ^13^C NMR: δ_c_ 36.60 CH_2_ aliphatic, (115.25, 115.40, 121.03, 124.95, 128.49, 132.78, 134.98, 157.37) C of benzene, (113.12, 120.98, 129.19, 134.99, 150.12) C of benzimidazole, 142.5 CH aliphatic, 158.96 C of ketone, 164.73 C of amide. ESI–MS (m/z) [M+1]^+^ 406.01; Anal. Calcd. for C_22_H_16_FN_3_O_2_S: C, 65.17; H, 3.98; F, 4.69; N, 10.36; O, 7.89; S, 7.91 Found: C, 65.14; H, 3.95; F, 4.63; N, 10.37; O, 7.81; S, 7.85.

#### *2*-*(1*-*Benzoyl*-*1H*-*benzo[d]imidazol*-*2*-*ylthio)*-*N*-*(2*-*chlorophenyl)acetamide***(4)**

Peach colored crystals, yield 82%, mp 137–140 °C, R_f_ 0.73 (*n*-hexane:ethylacetate 6:4); IR (ν_max_, cm-1): 3434 N–H str. for 2° amide, 2986 N–H str. for imidazole, 1694 C=O str. for 2° amide, 841 C–S str. of thiol. ^1^H NMR: δ_H_ 4.34 (s, 2H of methylene), 7.08–7.97 (m, 13H, aromatic), 10.06 (s, NH of 2° amide. ^13^C NMR: δ_c_ 36.72 CH_2_ aliphatic, (124.83, 125.42, 127.66, 128.50, 129.21, 129.41, 130.73, 132.79, 133.65, 134.74) C of benzene, (118.23, 123.14, 133.89, 143.00, 154.08) C of benzimidazole, 142.5 CH aliphatic, 166.94 C of ketone, 167.67 C of amide. ESI–MS (m/z) [M+1]^+^ 422.76; Anal. Calcd. for C_22_H_16_ClN_3_O_2_S: C, 62.63; H, 3.82; Cl, 8.40; N, 9.96; O, 7.58; S, 7.60 Found: C, 62.66; H, 3.78; Cl, 8.38; N, 9.97; O, 7.52; S, 7.63.

#### *2*-*(1*-*Benzoyl*-*1H*-*benzo[d]imidazol*-*2*-*ylthio)*-*N*-*(3*-*chlorophenyl)acetamide***(5)**

Cream colored crystals, yield 87%, mp 132–135 °C, R_f_ 0.59 (*n*-hexane:ethylacetate 6:4); IR (ν_max_, cm^−1^): 3406 N–H str. for 2° amide, 2977 N–H str. for imidazole, 1637 C=O str. for 2° amide, 781 C–Cl str. of monochlorinated compound, 740 C–S str. of thiol. ^1^H NMR: δ_H_ 4.49 (s, 2H of methylene), 7.12–7.83 (m, 13H, aromatic), 11.00 (s, NH of 2° amide). ^13^C NMR: δ_c_ 36.39 CH_2_ aliphatic, (117.52, 118.58, 128.50, 129.20, 133.07, 135.91, 135.99) C of benzene, (113.47, 123.27, 130.47, 140.15, 149.85) C of benzimidazole, 142.5 CH aliphatic, 165.88 C of amide. ESI–MS (m/z) [M+1]^+^ 422.79; Anal. Calcd. for C_22_H_16_ClN_3_O_2_S: C, 62.63; H, 3.82; Cl, 8.40; N, 9.96; O, 7.58; S, 7.60 Found: C, 62.64; H, 3.80; Cl, 8.36; N, 9.98; O, 7.54; S, 7.59.

#### *2*-*(1*-*Benzoyl*-*1H*-*benzo[d]imidazol*-*2*-*ylthio)*-*N*-*(2,5*-*dichlorophenyl)acetamide***(6)**

Yellow crystals, yield 79%, mp 138–140 °C, R_f_ 0.73 (*n*-hexane:ethylacetate 6:4); IR (ν_max_, cm^−1^): 3451 N–H str. for 2° amide, 3054 N–H str. for imidazole, 1690 C=O str. for 2° amide, 786 C–S str. of thiol, 710 C–S str. of polychlorinated compound. ^1^H NMR: δ_H_ 4.46 (s, 2H of methylene), 6.93–8.03 (m, 12H, aromatic), 10.56 (s, NH of 2° amide). ^13^C NMR: δ_c_ 35.83 CH_2_ aliphatic, (123.32, 125.67, 128.76, 129.04, 129.63, 130.81, 131.57, 132.01, 133.66, 136.40) C of benzene, (118.20, 123.84, 130.72, 142.94, 149.85) C of benzimidazole, 166.86 C of ketone, 167.24 C of amide. ESI–MS (m/z) [M+1]^+^ 456.17; Anal. Calcd. for C_22_H_15_Cl_2_N_3_O_2_S: C, 57.90; H, 3.31; Cl, 15.54; N, 9.21; O, 7.01; S, 7.03 Found: C, 57.86; H, 3.34; Cl, 15.48; N, 9.17; O, 7.04; S, 6.99.

#### *2*-*(1*-*Benzoyl*-*1H*-*benzo[d]imidazol*-*2*-*ylthio)*-*N*-*(2*-*bromophenyl)acetamide***(7)**

Brownish white crystals, yield 76%, mp 142–144 °C, R_f_ 0.61 (*n*-hexane:ethylacetate 6:4); IR (ν_max_, cm^−1^): 3463 N–H str. for 2° amide, 3052 N–H str. for imidazole, 1696 C=O str. for 2° amide, 727 C–S str. of thiol. ^1^H NMR: δ_H_ 4.46 (s, 2H of methylene), 6.93–8.03 (m, 13H aromatic), 10.14 (s, NH of 2° amide). ^13^C NMR: δ_c_ 36.69 CH_2_ aliphatic, (122.19, 124.31, 126.60, 128.05, 128.50, 129.21, 132.64, 133.91, 135.97) C of benzene, (118.32, 123.13, 130.72, 143.05, 153.97) C of benzimidazole, 166.78 C of ketone, 167.67 C of amide. ESI–MS (m/z) [M+1]^+^ 467.21; Anal. Calcd. for C_22_H_16_BrN_3_O_2_S: C, 56.66; H, 3.46; Br, 17.13; N, 9.01; O, 6.86; S, 6.88 Found: C, 56.61; H, 3.42; Br, 17.07; N, 9.06; O, 6.79; S, 6.85.

#### *2*-*(1*-*Benzoyl*-*1H*-*benzo[d]imidazol*-*2*-*ylthio)*-*N*-*(3*-*bromophenyl)acetamide***(8)**

Yellow crystals, yield 83%, mp 146–148 °C, R_f_ 0.63 (*n*-hexane:ethylacetate 6:4); IR (ν_max_, cm^−1^): 3474 N–H str. for 2° amide, 3173 N–H str. for imidazole, 1667 C=O str. for 2° amide, 822 C–H out of plane bending, 720 C–S str. of thiol, 659 C–Br str. aromatic. ^1^H NMR: δ_H_ 4.32 (s, 2H of methylene), 7.11–8.08 (m, 13H aromatic), 8.09 (s, NH of 2° amide). ^13^C NMR: δ_c_ 36.02 CH_2_ aliphatic, (117.82, 121.18, 121.41, 125.98, 127.38, 129.09, 129.29, 139.77, 140.54) C of benzene, (113.88, 121.59, 130.73, 139.30, 150.11) C of benzimidazole, 166.86 C of ketone, 170.46 C of amide. ESI–MS (m/z) [M+1]^+^ 467.19; Anal. Calcd. for C_22_H_16_BrN_3_O_2_S: C, 56.66; H, 3.46; Br, 17.13; N, 9.01; O, 6.86; S, 6.88 Found: C, 56.63; H, 3.39; Br, 17.09; N, 9.03; O, 6.83; S, 6.82.

#### *2*-*(1*-*Benzoyl*-*1H*-*benzo[d]imidazol*-*2*-*ylthio)*-*N*-*(4*-*bromophenyl)acetamide***(9)**

Light yellow crystals, yield 72%, mp 162–165 °C, R_f_ 0.81 (*n*-hexane:ethylacetate 6:4); IR (ν_max_, cm^−1^): 3451 N–H str. for 2° amide, 3055 N–H str. for imidazole, 1710 C=O str for 2° amide, 742 C–S str. of thiol, 623 C–Br str. aromatic. ^1^H NMR: δ_H_ 4.43 (s, 2H of methylene), 7.25–7.61 (m, 13H aromatic), 10.85 (s, NH of 2° amide). ^13^C NMR: δ_c_ 36.37 CH_2_ aliphatic, (115.15, 121.03, 128.49, 129.21, 136.74, 136.84, 136.791) C of benzene, (113.56, 122.82, 131.58, 138.10, 149.85) C of benzimidazole, 165.81 C of amide. ESI–MS (m/z) [M+1]^+^ 467.10; Anal. Calcd. for C_22_H_16_BrN_3_O_2_S: C, 56.66; H, 3.46; Br, 17.13; N, 9.01; O, 6.86; S, 6.88 Found: C, 56.59; H, 3.41; Br, 17.16; N, 8.96; O, 6.79; S, 6.78.

#### *2*-*(1*-*Benzoyl*-*1H*-*benzo[d]imidazol*-*2*-*ylthio)*-*N*-*(3*-*nitrophenyl)acetamide***(10)**

Dull cream colored crystals, yield 78%, mp 129–131 °C, R_f_ 0.61 (*n*-hexane:ethylacetate 6:4); IR (ν_max_, cm^−1^): 3470 N–H str. for 2° amide, 3007 N–H str. for imidazole, 1707 C=O str. for 2° amide, 1526 asymm. str. of aromatic nitro group, 1317 symm. str. of aromatic nitro group, 716 C–S str. of thiol. ^1^H NMR: δ_H_ 4.46 (s, 2H of methylene), 7.21–8.31 (m, 13H aromatic), 10.83 (s, NH of 2° amide). ^13^C NMR: δ_c_ 43.41 CH_2_ aliphatic, (125.32, 127.73, 128.48, 128.52, 129.21, 130.33, 130.79) C of benzene, (113.46, 118.35, 130.31, 132.81, 147.96) C of benzimidazole, 167.25 C of amide. ESI–MS (m/z) [M+1]^+^ 433.09; Anal. Calcd. for C_22_H_16_N_4_O_4_S: C, 61.10; H, 3.73; N, 12.96; O, 14.80; S, 7.41 Found: C, 61.03; H, 3.78; N, 12.89; O, 14.77; S, 7.35.

#### *2*-*(1*-*Benzoyl*-*1H*-*benzo[d]imidazol*-*2*-*ylthio)*-*N*-*(4*-*chloro*-*2*-*nitrophenyl)acetamide***(11)**

Orange crystals, yield 83%, mp 89–91 °C, R_f_ 0.80 (*n*-hexane:ethylacetate 6:4); IR (ν_max_, cm^−1^): 3481 N–H str. for 2° amide, 3030 N–H str. for imidazole, 1701 C=O str. for 2° amide, 1568 asymm. str. of aromatic nitro group, 1334 symm. str. of aromatic nitro group, 815 C–S str. of thiol, 704 C–Cl str. of monochlorinated aromatic compound. ^1^H NMR: δ_H_ 4.46 (s, 2H of methylene), 7.09–8.11 (m, 12H aromatic), 10.91 (s, NH of 2° amide). ^13^C NMR: δ_c_ 36.68 CH_2_ aliphatic, (123.96, 124.22, 129.04, 129.19, 129.94, 130.15, 130.64, 132.34, 133.96, 134.09, 135.49, 141.60) C of benzene, (114.36, 123.07, 130.73, 141.11, 153.53) C of benzimidazole, 167.61 C of amide. ESI–MS (m/z) [M+1]^+^ 467.76; Anal. Calcd. for C_22_H_15_ClN_4_O_4_S: C, 56.59; H, 3.24; Cl, 7.59; N, 12.00; O, 13.71; S, 6.87 Found: C, 56.51; H, 3.21; Cl, 7.53; N, 11.94; O, 13.76; S, 6.81.

#### *2*-*(1*-*Benzoyl*-*1H*-*benzo[d]imidazol*-*2*-*ylthio)*-*N*-*p*-*tolylacetamide***(12)**

Dull yellow crystals, yield 81%, mp 175–178 °C, R_f_ 0.71 (*n*-hexane:ethylacetate 6:4); IR (ν_max_, cm^−1^): 3361 N–H str. for 2° amide, 3166 N–H str. for imidazole, 2958 CH_3_ asymm. str. of Ar-CH_3_, 1695 C=O str. for 2º amide, 809 C–H out of plane bending of 1, 4- disubstituted benzene ring, 706 C–S str. of thiol. ^1^H NMR: δ_H_ 4.46 (s, 2H of methylene), 7.10–7.97 (m, 13H aromatic), 10.80 (s, NH of 2° amide). ^13^C NMR: δ_c_ 20.39 C of methyl, 36.63 CH_2_ aliphatic, (120.39, 128.49, 128.89, 129.20, 129.99, 130.25, 131.36, 132.65, 132.78, 136.03) C of benzene, (113.17, 123.11, 130.72, 136.09, 150.18) C of benzimidazole, 167.24 C of amide. ESI–MS (m/z) [M+1]^+^ 467.76; Anal. Calcd. for C_23_H_19_N_3_O_2_S: C, 68.81; H, 4.77; N, 10.47; O, 7.97; S, 7.99 Found: C, 68.86; H, 4.68; N, 10.37; O, 7.91; S, 7.94.

#### *2*-*(1*-*Benzoyl*-*1H*-*benzo[d]imidazol*-*2*-*ylthio)*-*N*-*(2,6*-*dimethylphenyl)acetamide***(13)**

Light yellow crystals, yield 73%, mp 166–168 °C, R_f_ 0.47 (*n*-hexane:ethylacetate 6:4); IR (ν_max_, cm^−1^): 3446 N–H str. for 2° amide, 3062 N–H str. for imidazole, 2979 CH_3_ asymm. str. of Ar-CH_3_, 1714 C=O str. for 2° amide, 740 C–H bending of trisubstituted benzene ring, 656 C–S str. of thiol. ^1^H NMR: δ_H_ 4.33 (s, 2H of methylene), 2.10–2.50 (m, 6H of methyl), 7.02–7.51 (m, 12H aromatic), 9.85 (s, NH of 2° amide). ^13^C NMR: δ_c_ (17.96, 18.17) C of two methyl, 35.15 CH_2_ aliphatic, (123.29, 126.47, 128.37, 128.51, 128.90, 129.22, 132.80, 134.70, 138.18) C of benzene, (113.91, 122.03, 130.74, 138.47, 149.81) C of benzimidazole, 167.27 C of amide. ESI–MS (m/z) [M+1]^+^ 416.37; Anal. Calcd. for C_24_H_21_N_3_O_2_S: C, 69.37; H, 5.09; N, 10.11; O, 7.70; S, 7.72 Found: C, 69.39; H, 5.13; N, 10.03; O, 7.64; S, 7.75.

#### *2*-*(1*-*Benzoyl*-*1H*-*benzo[d]imidazol*-*2*-*ylthio)*-*N*-*(3*-*methoxyphenyl)acetamide***(14)**

Light brown crystals, yield 74%, mp 170–172 °C, R_f_ 0.59 (*n*-hexane:ethylacetate 6:4); IR (ν_max_, cm^−1^): 3454 N–H str. for 2° amide, 3131 C–H str. of aralkyl ether, 3070 N–H str. for imidazole, 1526 N–H in plane bending of secondary amide, 1705 C=O str. for 2° amide, 1273 C–O–C asymm. str. of aralkyl ether, 1119 C–O–C symm. str. of aralkyl ether, 706 C–S str. of thiol. ^1^H NMR: δ_H_ 4.61 (s, 2H of methylene), 7.34–7.99 (m, 13H aromatic), 10.53 (s, NH of 2° amide). ^13^C NMR: δ_c_ 36.25 CH_2_ aliphatic, 63.11 C of methoxy, (113.61, 117.50, 128.76, 129.35, 130.22, 130.46, 133.58, 137.20, 140.18, 166.15) C of benzene, (113.70, 123.23, 130.69, 137.40, 149.72) C of benzimidazole, 168.67 C of amide. ESI–MS (m/z) [M+1]^+^ 418.19; Anal. Calcd. for C_23_H_19_N_3_O_3_S: C, 66.17; H, 4.59; N, 10.07; O, 11.50; S, 7.68 Found: C, 66.07; H, 4.53; N, 10.12; O, 11.43; S, 7.57.

#### *2*-*(1*-*Benzoyl*-*1H*-*benzo[d]imidazol*-*2*-*ylthio)*-*N*-*(4*-*chlorophenyl)acetamide***(15)**

Creamish yellow crystals, yield 81%, mp 158–160 °C, R_f_ 0.75 (*n*-hexane:ethylacetate 6:4); IR (ν_max_, cm^−1^): 3405 N–H str. for 2° amide, 3105 N–H str. for imidazole, 1653 C=O str. of secondary amide, 1536 N–H in plane bending of secondary amide, 741 C–Cl str. of monochlorinated aromatic compound, 624 C–S str. of thiol. ^1^H NMR: δ_H_ 4.64 (s, 2H of methylene), 7.36–7.70 (m, 13H aromatic), 11.06 (s, NH of 2° amide). ^13^C NMR: δ_c_ 36.64 CH_2_ aliphatic, (113.18, 120.75, 124.67, 127.25, 128.64, 133.32, 137.55, 150.05) C aromatic, 165.07 C of amide. ESI–MS (m/z) [M+1]^+^ 422.01; Anal. Calcd. for C_22_H_16_ClN_3_O_2_S: C, 62.63; H, 3.82; Cl, 8.40; N, 9.96; O, 7.58; S, 7.60 Found: C, 62.53; H, 3.75; Cl, 8.44; N, 9.86; O, 7.53; S, 7.57.

#### *2*-*(1*-*Benzoyl*-*1H*-*benzo[d]imidazol*-*2*-*ylthio)*-*N*–*o*-*tolylacetamide***(16)**

Dark brown crystals, yield 89%, mp 102–105 °C, R_f_ 0.76 (*n*-hexane:ethylacetate 6:4); IR (ν_max_, cm^−1^): 3332 N–H str. for 2° amide, 3014 N–H str. for imidazole, 2915 CH_3_ asymm. str. of Ar-CH_3_, 2363 CH_3_ symm. str. of Ar-CH_3_, 1679 C=O str. for 2° amide, 845 C–H out of plane bending of disubstituted benzene ring, 687 C–S str. of thiol. ^1^H NMR: δ_H_ 4.44 (s, 2H of methylene), 7.13–7.98 (m, 13H aromatic), 11.02 (s, NH of 2° amide). ^13^C NMR: δ_c_ 36.17 CH_2_ aliphatic, (11.91, 113.09, 124.37, 128.99, 129.04, 129.19, 129.58, 131.88, 133.42, 165.92) C of benzene, (113.21, 123.62, 130.71, 136.36, 149.83) C of benzimidazole, 168.67 C of amide. ESI–MS (m/z) [M+1]^+^ 402.16; Anal. Calcd. for C_23_H_19_N_3_O_2_S: C, 68.81; H, 4.77; N, 10.47; O, 7.97; S, 7.99 Found: C, 68.85; H, 4.70; N, 10.36; O, 7.92; S, 7.90.

#### *2*-*(1*-*Benzoyl*-*1H*-*benzo[d]imidazol*-*2*-*ylthio)*-*N*-*(2*-*methoxyphenyl)acetamide***(17)**

Light brown semisolid, yield 75%, mp—not determined (hygroscopic), R_f_ 0.59 (*n*-hexane:ethylacetate 6:4); IR (ν_max_, cm^−1^): 3466 N–H str. for 2° amide, 3033 C–H str. of aralkyl ether, 3077 N–H str. for imidazole, 1595 N–H in plane bending of secondary amide, 1705 C=O str. for 2° amide, 1247 C–O–C asymm. str. of aralkyl ether, 1022 C–O–C symm. str. of aralkyl ether, 718 C–S str. of thiol. ^1^H NMR: δ_H_ 4.43 (s, 2H of methylene), 6.99–8.03 (m, 13H aromatic), 10.54 (s, NH of 2° amide). ^13^C NMR: δ_c_ 35.84 CH_2_ aliphatic, 55.71 C of methoxy, (114.83, 122.22, 123.13, 125.63, 126.86, 129.20, 129.64, 131.56, 134.45, 164.92) C of benzene, (113.73, 123.29, 130.73, 136.86, 151.37) C of benzimidazole, 167.25 C of amide. ESI–MS (m/z) [M+1]^+^ 418.23; Anal. Calcd. for C_23_H_19_N_3_O_3_S: C, 66.17; H, 4.59; N, 10.07; O, 11.50; S, 7.68 Found: C, 66.08; H, 4.51; N, 10.03; O, 11.43; S, 7.72.

#### *2*-*(1*-*Benzoyl*-*1H*-*benzo[d]imidazol*-*2*-*ylthio)*-*N*-*(4*-*methoxyphenyl)acetamide***(18)**

Dark brown semisolid, yield 79%, mp—not determined (hygroscopic), R_f_ 0.67 (*n*-hexane:ethylacetate 6:4); IR (ν_max_, cm^−1^): 3326 N–H str. for 2° amide, 2972 C–H str. of aralkyl ether, 2606 N–H str. for imidazole, 1557 N–H in plane bending of secondary amide, 1702 C=O str. for 2° amide, 1230 C–O–C asymm. str. of aralkyl ether, 1022 C–O–C symm. str. of aralkyl ether, 710 C–S str. of thiol. ^1^H NMR: δ_H_ 4.33 (s, 2H of methylene), 7.12–8.03 (m, 13H aromatic), 10.61 (s, NH of 2° amide). ^13^C NMR: δ_c_ 36.00 CH_2_ aliphatic, 55.16 C of methoxy, (114.06, 127.53, 128.34, 129.19, 131.67, 132.25, 134.97, 155.53) C of benzene, (113.56, 122.22, 131.29, 139.39, 150.71) C of benzimidazole, 167.68 C of amide. ESI–MS (m/z) [M+1]^+^ 418.19; Anal. Calcd. for C_23_H_19_N_3_O_3_S: C, 66.17; H, 4.59; N, 10.07; O, 11.50; S, 7.68 Found: C, 66.11; H, 4.61; N, 10.03; O, 11.45; S, 7.74.

#### *2*-*(1*-*Benzoyl*-*1H*-*benzo[d]imidazol*-*2*-*ylthio)*-*N*-*m*-*tolylacetamide***(19)**

Cream colored semisolid, yield 89%, mp—not determined (hygroscopic), R_f_ 0.69 (*n*-hexane:ethylacetate 6:4); IR (ν_max_, cm^−1^): 3406 N–H str. for 2° amide, 2957 N–H str. for imidazole, 2957 CH_3_ asymm. str. of Ar-CH_3_, 2893 CH_3_ symm. str. of Ar-CH_3_, 1633 C=O str. for 2° amide, 701 C–S str. of thiol. ^1^H NMR: δ_H_ 2.32 (s, 3H of methyl), 4.47 (s, 2H of methylene), 7.18–7.98 (m, 13H aromatic), 10.72 (s, NH of 2° amide). ^13^C NMR: δ_c_ 36.33 CH_2_ aliphatic, 21.17 C of methyl, (119.60, 122.72, 124.28, 128.28, 128.72, 129.34, 131.42, 134.93, 137.94, 138.69) C of benzene, (113.43, 122.94, 130.73, 139.06, 149.93) C of benzimidazole, 142.5 CH aliphatic, 164.79 C of ketone, 167.25 C of amide. ESI–MS (m/z) [M+1]^+^ 402.23; Anal. Calcd. for C_23_H_19_N_3_O_2_S: C, 68.81; H, 4.77; N, 10.47; O, 7.97; S, 7.99 Found: C, 68.76; H, 4.81; N, 10.41; O, 7.93; S, 7.92.

#### *2*-*(1*-*Benzoyl*-*1H*-*benzo[d]imidazol*-*2*-*ylthio)*-*N*-*(2,4*-*dimethylphenyl)acetamide***(20)**

Peach colored semisolid, yield 81%, mp- not determined (hygroscopic), R_f_ 0.74 (*n*-hexane:ethylacetate 6:4); IR (ν_max_, cm^−1^): 3406 N–H str. for 2° amide, 3134 N–H str. for imidazole, 2919 CH_3_ asymm. str. of Ar-CH_3_, 2876 CH_3_ symm. str. of Ar-CH_3_, 1638 C=O str. for 2° amide, 702 C–S str. of thiol. ^1^H NMR: δ_H_ 4.48 (s, 2H of methylene), 2.16–2.28 (m, 6H of methyl), 6.99–7.97 (m, 12H aromatic), 10.01 (s, NH of 2° amide). ^13^C NMR: δ_c_ (17.74, 20.55) c of two methyl, 35.97 CH_2_ aliphatic, (120.32, 126.55, 129.39, 129.68, 130.73, 131.35, 134.26, 134.37, 134.45, 134.53) C of benzene, (113.27, 123.23, 130.77, 135.00, 150.04) C of benzimidazole, 167.24 C of amide. ESI–MS (m/z) [M+1]^+^ 416.19; Anal. Calcd. for C_24_H_21_N_3_O_2_S: C, 69.37; H, 5.09; N, 10.11; O, 7.70; S, 7.72 Found: C, 69.40; H, 5.01; N, 10.03; O, 7.62; S, 7.67.

## Antimicrobial activity evaluation

### Determination of MIC

The in vitro antimicrobial activity of the synthesized derivatives was evaluated against *Escherichia coli, Salmonella typhi* (Gram-negative bacteria); *Bacillus subtilis*, *Staphylococcus aureus*, *Bacillus cereus*, (Gram-positive bacteria); *C. albicans* and *A. niger* (fungal strains) using tube dilution method [33]. Cefadroxil and fluconazole were used as standard antibacterial and antifungal drugs respectively. The stock solutions of 100 µg/ml concentration were prepared in dimethyl sulfoxide for both test and standard drugs. Both the standard and test compounds were serially diluted in double strength nutrient broth I.P. for bacteria and Sabouraud dextrose broth I.P. for fungi [34]. The bacterial cultures were incubated for a period of 24 h at 37 ± 2 °C. The incubation time for *C. albicans* was 48 h at 37 ± 2 °C and for *A. niger* was 7 days at 25 ± 2 °C. The results of antimicrobial activity were stated in terms of minimum inhibitory concentration (MIC).

### Determination of MBC/MFC

The minimum bactericidal concentration (MBC) and minimum fungicidal concentration (MFC) of the synthesized benzimidazole derivatives was determined by subculturing 100 µl of culture from each tube that remained clear in MIC determination onto sterilized petri-plates containing fresh agar medium. The petri-plates were incubated and analyzed for microbial growth visually [35].

### In vitro antitubercular activity evaluation

The antimycobacterial activity of synthesized compounds was performed in three level safety laboratories at National Centre of Fungal Taxonomy (NCFT), New Delhi in association with HIHT University, Jolly Grant, Dehradun (U.K). The preserved strains of *M. tuberculosis* viz., Mycobacterium sensitive to streptomycin (S), isoniazid (H), rifampin (R) and pyrazinamide (PZA)-H37Rv (NCFT/TB/537) was used in order to assess the antimycobacterial activity of the compounds. Middle brook 7H10 agar (Becton–Dickinson Company (DifcoTM), 7 Loveton Circle, Sparks, Maryland, USA; Lot No. 8175150) supplemented with oleic acid-albumin catalase (OADC) (Becton–Dickinson Company Lot 8136781) for antimycobacterial activity was used to revive and culture the mycobacteria for sensitivity testing. Streptomycin (500 mg), standard antimycobacterial drug, was obtained as gift sample from Shalina Laboratories Pvt. Ltd., Navi Mumbai, Maharashtra.

### Preparation of the drugs/compounds dilutions

Each of the synthesized derivatives was dissolved in DMSO to obtain a concentration of 50 µg/ml and diluted further to a concentration of 25 and 12.5 µg/ml. Similarly, stock solution of 50 µg/ml concentration was prepared for standard antitubercular drug, streptomycin and diluted further to 25 µg/ml in order to check the antitubercular activity.

### Preparation of growth media

It was prepared by adding dehydrated medium (19 g) to purified water (900 ml) containing glycerol (l5 ml). The mixture was stirred well to dissolve and autoclaved at 121 °C for 10 min. Oleic acid-albumin catalase (100 ml) was aseptically added to the medium after cooling to 45 °C. No adjustment for pH was made.

### Preparation of inoculum for drug sensitivity testing

Preserved strains of *M. tuberculosis* viz, mycobacterium sensitive to S, H, R and PZA-H37Rv (NCFT/TB/537) was revived on Middle brook 7H10 agar, prior to antituberculosis susceptibility testing. Cells were scraped from freshly grown colonies (3 weeks old) on Middle brook 7H10 plates and introduced into saline (10 ml). Bacterial suspensions with 0.5 McFarland standard turbidity equivalents to 10^8^ CFU were prepared by dilution with saline. The mixture was vortexed for 30 s in a glass bottle containing glass beads and the particles were allowed to settle [36].

### Random screening of the isolated compounds for antitubercular activity (Alamar-blue assay)

The antimycobacterial activity of compounds was assessed against mycobacterium sensitive to S, H, R and PZA-H37Rv (NCFT/TB/537); using the microplate alamar blue assay (MABA) [37]. This methodology is nontoxic, uses a thermally-stable reagent and is suitable for random screening of the antimycobacterial activity. Briefly, 200 μl of sterile deionized water was added to all outer-perimeter wells of sterile 96 well plates to minimize evaporation of the medium in the test wells during incubation. The 96 well plates received 100 μl of the Middle brook 7H9 broth (having loopful inoculum of bacteria-10^8^ CFU) and different dilutions of the respective compounds were made directly on the plate. Plates were covered and sealed with parafilm and incubated at 37 °C for 5 days. After this time, 25 μl of a freshly prepared 1:1 mixture of alamar blue reagent and 10% tween 80 was added to the plate and incubated for 24 h. A blue color in the well was interpreted as no bacterial growth (antimycobacterial activity), and a pink color was scored as growth.

### Bioassay protocol for susceptibility tests of the compounds by well diffusion method

The well diffusion method was used to determine susceptibility [36, 38]. The agar well diffusion method [39] was modified and Middle brook 7H10 agar medium was used. The culture medium was inoculated with loopful bacteria separately suspended in Middle brook 7H10 broth. Wells of 8 mm diameter were punched into agar and filled each well separately with 50 µg/ml of test compound and 25 µg/ml of standard drug. The petri-dishes were then left in the hood overnight to allow diffusion of the drug and then sealed with a carbon dioxide-permeable tape. These were then incubated at 37 °C in a carbon dioxide incubator for 4 weeks. The wells were flooded with alamar-blue dye in highly sterilized chamber and de-stained further to observe the zones of inhibition. The sensitivity of the strains to the compounds was determined by measuring the diameter of zones of inhibition (in millimeter) around the well.

### Determination of the minimum inhibitory concentration (MIC) by alamar blue assay

The compounds were serially diluted to determine the minimum inhibitory concentration of the drug in Middle brook 7H9 medium using microplate alamar blue assay [36, 40, 41]. The compounds which were found satisfactory by the above two methods at a maximum concentration of 50 µg/ml were diluted further to concentrations viz., 25, 12.5, 6.25, 3.125 and 1.56 µg/ml respectively. Similarly, streptomycin was further diluted to 25 µg/ml in order to check the antitubercular activity. The plates were covered and sealed with parafilm and incubated at 37 °C for 5 days. After this time, 25 μl of a freshly prepared 1:1 mixture of alamar blue reagent and 10% tween 80 was added to the plate and incubated for 24 h. A blue color in the well was interpreted as no bacterial growth (antimycobacterial activity) and appearance of pink color was determined as growth. The MIC is defined as the lowest drug concentration which prevented a color change from blue to pink.

### In vivo antitubercular activity evaluation

The LD_50_ (lethal dose) and ED_50_ (optimum/effective dose) doses were determined for the active compounds in mice models infected with *Mycobacterium* H37Rv via ethical permission no., NCFT/EC/16/2313 assigned to Collaborative Research Group (CRG), NCFT, New Delhi, India.

### Enzyme assays for antitubercular activity

The compounds found potent in in vivo evaluation were assayed for inhibition of mycobacterial enzymes viz., isocitrate lyase, pantothenate synthetase and chorismate mutase.

### Mycobacterial isocitrate lyase (ICL) assay

Isocitrate lyase activity was assayed according to the protocol reported by Dixon and Kornberg (glyoxylate phenyl hydrazone formation) [42] at 10 μM of the compounds. Isoniazid was employed as a negative control (inhibition of 0%) and streptomycin sulphate (25 μg/kg) served as a positive control [43].

### Mycobacterial pantothenate synthetase (PS) assay

About 60 µl of the PS reagent, including NADH, pantoic acid, -alanine, ATP, phosphoenolpyruvate, MgCl_2_, myokinase, pyruvate kinase, and lactate dehydrogenase in buffer was added to each well of a 96-well plate. The compounds were then added to plates in 1 µl volumes. The reaction was initiated by the addition of 39 µl PS diluted in buffer. The final concentrations in the reaction contained 0.4 mM NADH, 5 mM pantoic acid, 10 mM MgCl_2_, 5 mM -alanine, 10 mM ATP, 1 mM potassium phosphoenolpyruvate, and 18 units/ml each of chicken muscle myokinase, rabbit muscle pyruvate kinase and rabbit muscle lactate dehydrogenase diluted in 100 mM HEPES buffer (pH 7.8), 1% DMSO, and 5 µg/ml PS in the final volume of 100 µl. The absorbance was measured using microplate reader at 340 nm after every 12 s for 120 s. Each plate had 16 control wells in the two outside columns, of which 12 contained the complete reaction mixture with a DMSO carrier control (full reaction) and four without PS. The per cent inhibition was calculated using the following formula: 100 × (1 − compound rate − background rate)/(full reaction rate − background rate) [44, 45].

### Mycobacterial chorismate mutase (CM) assay

Reaction volumes of 0.4 ml of chorismate (typically 1 mM) in 50 mM Tris HCl (pH 7.5), 0.5 mM EDTA, 0.1 mg/ml bovine serum albumin, and 10 mM -mercaptoethanol were incubated at 37 °C for 5 min. The reaction was started with the addition of 10 µl 5 pM of MtCM (i.e., 185 ng of CM equivalent to 12.5 nM final concentration of the dimer based on the molecular mass of 36,948 Da). The reaction was allowed to proceed at 37 °C and was terminated after 1–5 min with 0.4 ml 1 M HCl. After further incubation at 37 °C for 10 min, 0.8 ml 2.5 M NaOH was added to convert prephenate formed in the enzymatic reaction to phenyl pyruvate. The absorbance of phenylpyruvate chromophore was taken at 320 nm. A blank with no enzyme for every reaction was also set to account for the non-enzymatic conversion of chorismate to prephenate and enzyme was added after the addition of NaOH. The absorbance at 320 nm for the blank varied from 0.1 to 0.3, depending upon the concentration of chorismate and the duration of the reaction [46].

### In vitro anticancer screening

The in vitro cytotoxicity screening of the synthesized benzimidazole derivatives was assessed on MCF7 (human breast cancer) and HCT116 (human colorectal) cell line by Sulforhodamine-B (SRB) assay [47]. The results of anticancer activity were expressed as IC_50_ (amount of drug necessary to reduce the cell viability by 50%) and compared with the standard anticancer drugs, tamoxifen and 5-fluorouracil for MCF7 and HCT116 cell lines, respectively.

## Abbreviations

HCT116: human colorectal cell line
MCF7: human breast carcinoma cell line
MIC: minimum inhibitory concentration
MLC: minimum lethal concentration
MBC: minimum bactericidal concentration
MFC: minimum fungicidal concentration
IR: infrared spectroscopy
^1^HNMR: proton nuclear magnetic resonance
^13^CNMR: carbon nuclear magnetic resonance
*S. aureus*: *Staphylococcus aureus*
*B. subtilis*: *Bacillus subtilis*
*B. cereus*: *Bacillus cereus*
*S. typhi*: *Salmonella typhi*
*E. coli*: *Escherichia coli*
*C. albicans*: *Candida albicans*
*A. niger*: *Aspergillus niger*
